# A comprehensive hog plum leaf disease dataset for enhanced detection and classification

**DOI:** 10.1016/j.dib.2025.111311

**Published:** 2025-01-21

**Authors:** Sabbir Hossain Durjoy, Md. Emon Shikder, Mayen Uddin Mojumdar

**Affiliations:** Multidisciplinary Action Research Lab, Department of Computer Science and Engineering, Daffodil International University, Daffodil Smart City, Birulia, Dhaka 1216, Bangladesh

**Keywords:** Agricultural dataset, Machine learning, Precision farming, Disease detection

## Abstract

A comprehensive Hog plum leaf disease dataset is greatly needed for agricultural research, precision agriculture, and efficient management of disease. It will find applications toward the formulation of machine learning models for early detection and classification of disease, thus reducing dependency on manual inspections and timely interventions. Such a dataset provides a benchmark for training and testing algorithms, further enhancing automated monitoring systems and decision-support tools in sustainable agriculture. It enables better crop management, less use of chemicals, and more focused agronomical practices. This dataset will contribute to the global research being carried out for the advancement of disease-resistant plant strategy development and efficient management practices for better agricultural productivity along with sustainability. These images have been collected from different regions of Bangladesh. In this work, two classes were used: ‘**Healthy**’ and **‘Insect hole’**, representing different stages of disease progression. The augmentation techniques that involve flipping, rotating, scaling, translating, cropping, adding noise, adjusting brightness, adjusting contrast, and scaling expanded a dataset of 3782 images to 20,000 images. These have formed very robust deep learning training sets, hence better detection of the disease.

Specifications Table

**Table:** 

Subject	Agricultural Science, Computer Vision.
Specific subject area	Hog plum leaf disease detection, image classification, machine learning, deep learning, agricultural disease management*.*
Type of data	Images (.jpg) Raw, Filtered, Analysed
Data collection	Field surveys by expertise in different regions of Bangladesh captured 3782 high-quality images depicting Hog plum leaves in different health conditions. The dataset was further augmented to 20,000 images with different augmentation techniques that include flipping, rotation, translation, scaling, adding noise, adjusting brightness, adjusting contrast, and scaling in order to offer a more robust set for model training and evaluation.
Data source location	The data were collected from the following location: 1. Daffodil Smart City, Birulia, Savar, Dhaka, Bangladesh. (Latitude: 23° 52′ 37.35″ N, Longitude: 90° 19′ 18.29″ E) 2. The National Botanical Garden of Bangladesh, Mirpur, Dhaka - 1216. (Latitude: 23° 48′ 46.96″ N, Longitude: 90° 20′ 51.87″ E) 3. Zailla, Singair, Manikganj, Dhaka, Bangladesh. (Latitude: 3° 47′ 46.11″ N, Longitude: 90° 13′ 15.73″ E)
Data accessibility	Repository name: Mendeley Data Data identification number: 10.17632/yvtn2gp8zg.1 Direct URL to data: https://data.mendeley.com/datasets/yvtn2gp8zg/1
Related research article	None

## 1 Value of the Data

• The Hog plum leaf disease dataset presents the computer vision and machine learning models with the opportunity to effectively classify leaves between healthy and diseased classes, enabling improved plant health management through early intervention. This dataset helps in creating models to detect leaf diseases and improve plant health
• This dataset contains high-quality images labeled for healthy and insect-attacked leaves, thus providing a good backbone in training and testing disease classification models [3]. It has clear, high-quality images of both healthy and diseased leaves for training and testing.
• It gives researchers the power to design, train, and test algorithms so that they can differentiate between healthy and diseased leaves; this will allow real-time agricultural monitoring and decision-making tools. Researchers can use it to build algorithms that identify leaf diseases quickly and accurately.
• The dataset allows for algorithm benchmarking and invites comparative studies. These lead to a further step forward in leaf disease detection and image augmentations. This dataset allows comparison of methods and encourages better techniques for leaf disease detection [8].
• This is a dataset that promotes collaboration between plant pathology and computer vision fields, driving innovative applications of agricultural technology and sustainable farming practices. It supports teamwork between agriculture and technology experts to develop better solutions.

## 2 Background

Hog plum (*Spondias mombin*) is a tropical fruit tree that holds significant agricultural and economic value, particularly in tropical and subtropical regions. Renowned for its nutritional and medicinal properties, it has gained increasing acceptance in both domestic and international markets. Despite its global significance, there is a lack of comprehensive datasets focusing on Hog plum leaf diseases.

The Hog plum leaf disease dataset is a collection of 3782 original images representing different stages and conditions of leaves that have been categorized into two classes: ‘**Healthy**’ and **‘Insect Hole Spot’**. These images were acquired from different geographical locations in Bangladesh under varying environmental conditions and at various growth phases of the Hog plum trees, ensuring the wide and representative collection of disease manifestations.

The dataset presented will be very important to both researchers and practitioners in the field of precision agriculture [9]. From this dataset, researchers are allowed to contribute to and validate machine learning algorithms that best fit the detection and classification of Hog plum leaf diseases, which are important in disease management strategies where a farmer can identify issues in their tree at an early stage and take action on them in good time, therefore decreasing fruits loss and improving their potentials [15].

The dataset further allows collaboration and innovativeness on the side of agriculture, among other scientific fields. It will provide ground for exploring new approaches to the detection of diseases [10], allowing training in computer vision models that then can be integrated into advanced technologies for real farming applications [6]. This will not only serve to improve tree health management but also pave the way for sustainable agriculture. Such a dataset would, therefore, avail informed decisions to farmers and other players in the agriculture sector, hence building resilience and ensuring prosperity in the production of the Hog plum, therefore contributing towards the overall sustainability and productivity of the agricultural sector.

## 3 Data Description

The hog plum is a tropical fruit tree that is of nutritional and medicinal importance. It plays an important role in agriculture and industries of food manufacture. Its fruits are of great economic value in both local and international markets due to their high antioxidant content and multiple uses. Despite this, the plant remains prone to several diseases, especially of the leaves, which can seriously lower the yield and quality of the crop. This brings into focus the need for effective disease detection and management.

It is a rich dataset consisting of 3782 high-quality images of Hog plum leaves of two classes: Healthy and Insect Hole Spot. It was collected from different geographical regions of Bangladesh under different environmental conditions and hence captures the natural variations in lighting, leaf orientation, and disease symptoms. Techniques for augmentation, such as flipping, rotation, and scaling, were applied to this dataset to extend it to 20,000 images, thus making it more robust for training machine learning models [11]. This dataset represents all stages of leaf health conditions. The images were collected from different regions of Bangladesh and captured with a Xiaomi Redmi Note 10 Pro Max smartphone during field surveys carried out by the experts. These are pictures of natural variation in the cultivation of the Hog plum, both the diseased and healthy leaves, (see in Table 1) considering variation in lighting and backgrounds. Each of these images is shot with care in order to capture characteristic symptoms of leaf diseases that include discoloration, curling, necrosis, and deformation for comprehensive information.

**Table 1:** Hog plum leaf class wise overview.

Class Name	Description	Visualization
**Healthy**	Pictures of healthy Hog plum leaves, which depict the best condition of the plant [16]. Healthy Hog plum leaf present vibrant green color, uniform shape, and absence of disease	
**Insect Hole Spot**	The images of leaves affected by different types of leaf diseases that might include different shapes, such as discoloration, wilting, necrosis, or other deformed shapes that might point toward different forms of disease manifestation	

The Hog Plum Leaf Disease Dataset is unique in that there are currently no similar datasets specifically focused on *Spondias mombin*, the tropical fruit known as Hog Plum. Unlike more commonly studied crops, such as cotton, rice, or tomato, which have well-established disease detection datasets, the Hog Plum dataset addresses a gap in agricultural research [13]. This dataset is the first to focus specifically on Hog Plum leaves and their diseases.

Data collection was done keeping in mind the challenges that would, among others, involve different conditions of lighting, background noise, or other problems so high-quality, enlightening images can be provided. Proper representation of disease symptoms is highly crucial since correct training of machine learning models means such models are useful to build for automatically detecting diseases. It opens up avenues for early detection, hence effectively targeting treatments and reducing superfluous uses of pesticides, eventually improving crop health in general.

Class Details**:** There are mainly two classes in this dataset **healthy** and **Insect Hole Spot**

Hog plum a tropical fruit with increasing acceptance in both national and international fruit markets [1]. The Hog plum leaf disease dataset can be used in several agricultural and technological applications. From this dataset, through machine learning models, the researcher will be able to devise a number of systems that could identify and classify diseases into types for earlier detection and management of the disease outbreaks [12]. Hog plums (*Spondias mombin*) are nutrient-rich medicinal plants with antioxidants, valued in the food, agricultural, and health industries [2].

This dataset allows machine learning algorithms to grade disease prevalence and severity, hence guiding farmers in making informed decisions about targeted interventions in optimizing tree management practices.

The dataset would help in the development of a remote sensing and UAV-based monitoring system assessing the change in health at leaf level and possibly discriminating hotspots of disease to support proactive agriculture.

The high-resolution, field-collected Hog plum leaf disease dataset captures the manifestations of diseases under varying environmental conditions [14]. Such controlled-environment datasets are rather noted for their limitation to diversity. This dataset will add value by including images taken at different stages and under various conditions, hence helping construct robust and generalizable machine learning models for use in sustainable agriculture practices

Unique features of this dataset include its focus on real-world conditions, detailed class labeling, and augmentation diversity, making it a very valued benchmark for precision agriculture research and applications.

## 4 Experimental Design, Materials and Methods

### 4.1 Experimental design

The dataset for the training and testing of the machine learning models in disease detection was a Hog plum leaf image dataset. The images taken included healthy and diseased leaves at different stages of growth, various conditions, and other distinguishing factors. Preprocessing consisted of resampling the images at 800×800 pixels, followed by normalizing pixel values between 0 and 1, and purging duplicate and low-quality images.

The Hog Plum leaf images were collected through field surveys conducted in Bangladesh from June to October 2024. The collection sites were carefully selected to represent diverse environmental and cultivation conditions, ensuring the dataset's robustness and applicability. The geographic coordinates of the collection sites are as follows:

1. Daffodil Smart City, Savar, Dhaka (10 June 2024):
  **Latitude:** 23°52′37.35″N, **Longitude:** 90°19′18.29″E
2. National Botanical Garden, Mirpur, Dhaka (2 August 2024):
  **Latitude:** 23°48′46.96″N, **Longitude:** 90°20′51.87″E
3. Zailla, Singair, Manikganj (25 October 2024):
  **Latitude:** 23°47′46.11″N, **Longitude:** 90°13′15.73″E

Then, the stratification for training and validation was set at 80 % and 20 %, respectively. Deep learning models such as LeNet, VGG16, VGG19, Inception V3, ResNet-50, ResNet-101, EfficientNet, and DenseNet201 were trained with a batch size of 32 for 30 epochs, where an adaptive learning rate scheduler is used to have improved performance with the avoidance of overfitting [5].

**Fig. 1:**
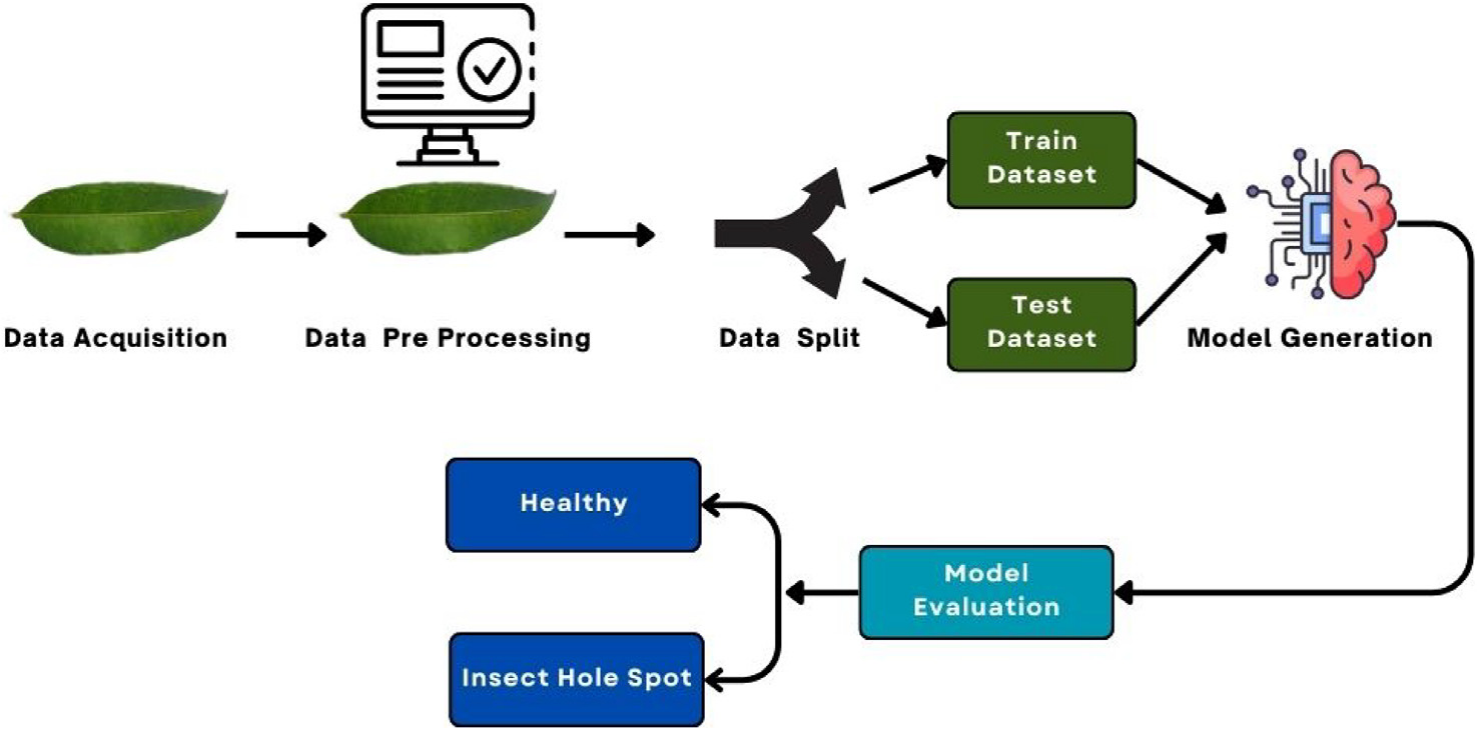
The method by which the diseases of Hog Plum leaf is evaluated.

In Fig. 1. The model gives an overall view of model performance in classifying images as **‘Healthy’** or **‘Insect Hole Spot’**. Progress on training was observed with the help of accuracy and loss curves to get an idea about the learning dynamics of models, such as whether any overfitting or underfitting problem persists. In view of this, the objective of the study was to perform modeling that would leaf performance not only on training data but also generalize well to new unseen images.

It described how the creation, pre-processing, and usage of the Hog plum leaf dataset were performed with due care in a structured manner to ensure that the deep learning models would actually result from it and perform the task of detection reliably and efficiently to help improve agricultural practices and crop health management.

### 4.2 Materials (Camera specification)

**Fig. 2:**
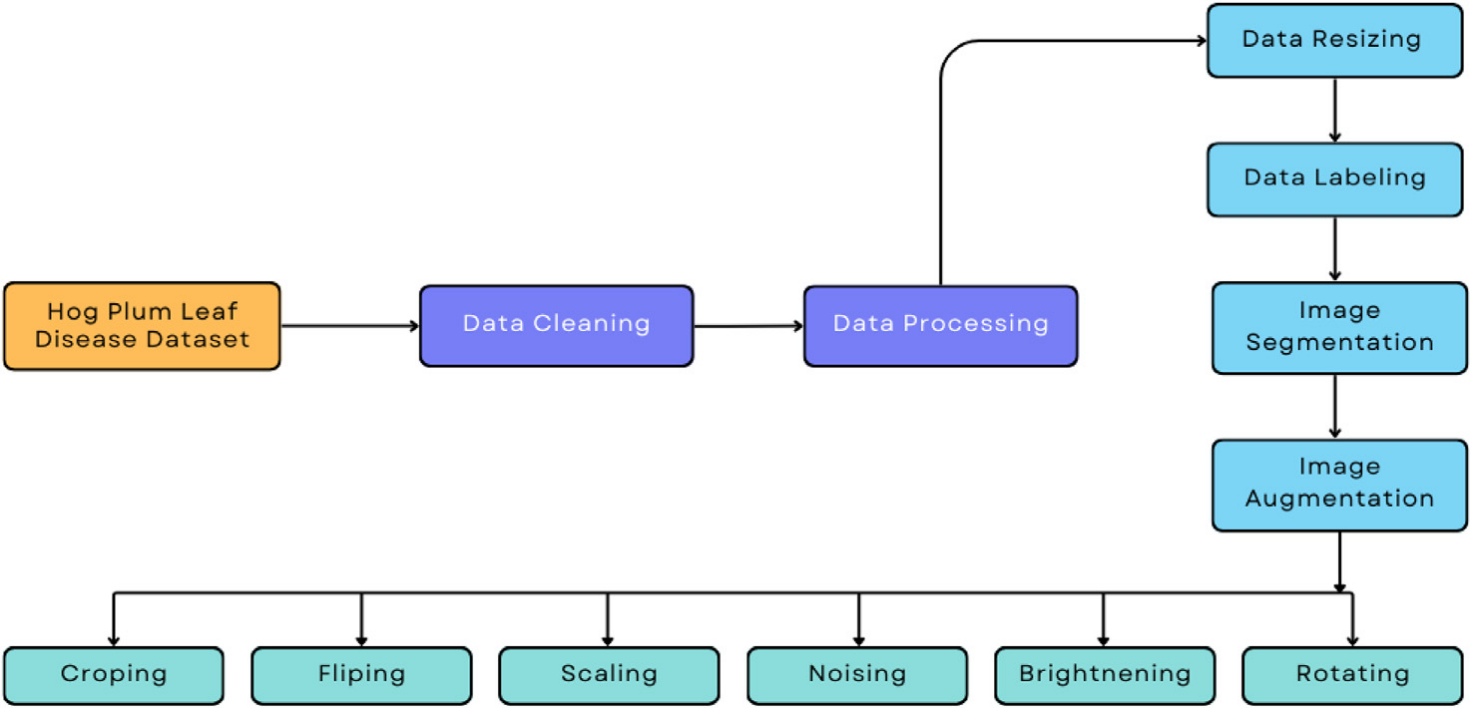
The proposed deep learning model's pre-processing stages.

Data acquisition was done by using the Redmi Note 10 Pro Max, having a 108 MP primary camera with an f/1.9 aperture. It made the capturing of higher-quality images in good and poor light conditions possible. Fine details and texture information necessary for healthy versus diseased Hog plum leaves were captured using the camera. An ultra-wide 8 MP lens with a field of view of 118° easily captured whole-leaf or multi-leaf shots, while the 2 MP macro lens was used to closely capture lesions, discoloration, and necrosis. The addition of a depth sensor added dimension to portrait shots to accurately represent leaf health from multiple angles (Fig. 2).

## 5 Methods

Artificial intelligence-based deep learning methods play a crucial role in disease detection by analysing large volumes of plant leaf images [4]. Carefully preparing and preprocessing the Hog plum leaf disease dataset to make the models more robust in machine learning was essential. Images were collected from field surveys in Bangladesh with agricultural experts, capturing healthy and diseased leaves under natural conditions to ensure symptoms of discoloration, necrosis, and deformation.

**Fig. 3:**
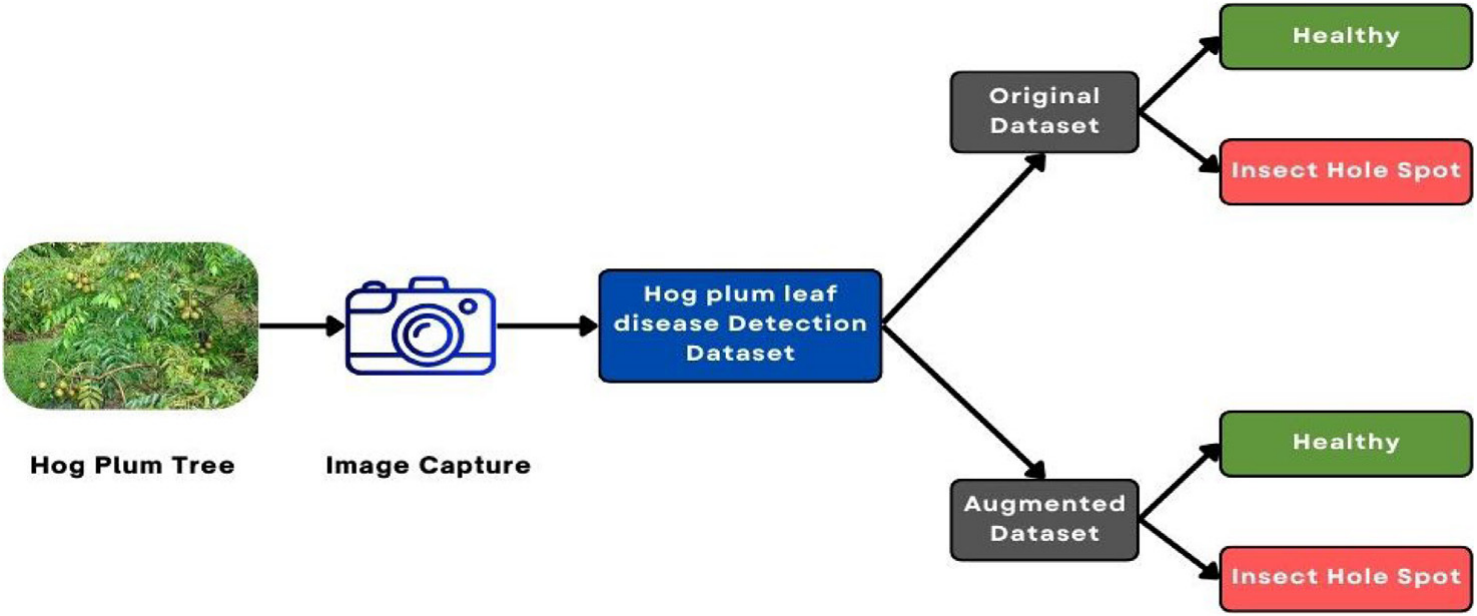
Dataset organization of Hog Plum leaf disease.

Preprocessing was necessary to unify the images by size, normalize the pixels' values (0–1), and facilitate faster model training. Low-quality and duplicate images were removed. Data augmentation techniques used included flipping, rotation up to 45°, scaling between 0.8 and 1.2, translation, cropping, noise addition, and brightness or contrast modifications, increasing the dataset to approximately 20,000 images, ensuring much better variation and adaptability to real-world settings (Fig. 3).

Model training was carried out using a batch size of 32 and an adaptive learning rate scheduler to keep the learning at an optimum and avoid overfitting. Training was run for 30 epochs, or, in the case of early stopping, if it reached that criterion, then the training stopped when performance on the validation set remained constant. Performance checks were performed on this model with a validation subset containing 20 % of the data.

This systematic process of data acquisition, preprocessing, augmentation, and training provided the deep learning models with ample opportunities to learn the most efficient and speedy detection methods of diseases in Hog plum leaves [7]. This approach of utilizing a combination of different image augmentations and strong methods for model training laid the foundation for a reliable tool that might begin to emerge for early disease detection to assist farmers and researchers in employing efficacious disease management practices.

### 5.1 Data annotation protocol

The annotation process was performed by a single expert agronomist, **Abdul Mannan Mojumdar** Former Additional Director in **Department of Agricultural Extension in Bangladesh**, who has extensive experience in plant disease diagnosis and classification. The steps followed were:

1. **Initial Screening:** Each image was checked for quality, ensuring that it was clear and captured the details of the disease. Low-quality or unclear images were excluded.
2. **Class Assignment:** Visible symptoms such as discoloration, necrosis, and other types of deformation thus assigned images to either one of two classes: **Healthy** and **Insect Hole Spot.**
3. **Verification:** After initial labeling, the annotations were reviewed to make sure accuracy and consistency were considered across the dataset.

This consistent and expert-driven annotation protocol thus ensures that the dataset is of high quality for use in machine learning-based disease detection and classification tasks.

### 5.2 Data augmentation

These enhancements are key to improving the robustness and generalization capability of the deep learning models when training on the Hog plum leaf disease dataset. Since there is always a challenge either of little availability of data or the need to perform well under diverse conditions in the real world, there exists a need to synthetically expand the training dataset using data augmentation techniques. Then, several transformations on the already available images enhanced this dataset to a total of about 20,000 images, enriching the pool of training data by a big margin.

The data augmentation approach is used to enhance variety and robustness in the Hog Plum Leaf Disease Dataset, thereby enhancing training and evaluation. Methods used here are flipping, rotation up to 45°, scaling from 0.8 to 1.2, translation, cropping, adding noise, and changing brightness and contrast. These transformations simulate real conditions and help the model to generalize on data it has never seen.

The dataset contains the original and augmented images as follows:

**Original Dataset:** This dataset contains 3782 images divided into two classes: **healthy** and **not_healthy leaves**.
**Augmented Dataset:** The augmented dataset is developed using augmentation techniques, and in total, it has around 20,000 images. The augmented images are kept in their respective classes, **healthy** and **not_healthy**, and are separated for model training.

It is worth noting that, although the augmented images give more variety and robustness during training, they are not part of the main dataset. The Original Dataset (containing 3782 images) is the main data, and the augmented images are used to support model development without artificially inflating the dataset for public release.

Other edits included cropping, which offered different views by focusing on specific parts of the leaves. Besides adding noise as environmental factors for things like dirt or effects caused by cameras, changes in brightness and contrast were made to simulate changing lighting conditions when collecting the data. This was achieved by altering the brightness levels between 0.7 and 1.3. Such edits were important in ascertaining that the models would generalize well in images taken under different intensities of sunlight and shade.

All these augmentation methods ensured that the variations captured by the dataset were as broad as possible, and therefore, this dataset would act as an influential tool in training deep learning models with enhanced generalization capability. Augmentation helps to avoid overfitting, a scenario where a model may train well on the training data but fails to generalize when it comes to unseen data, by showing the model more variable training examples. The broad approach applied let the development of machine learning models that, even with completely new images or slightly modified ones, had a better preparation to recognize and classify Hog plum leaf diseases.

**Fig. 4:**
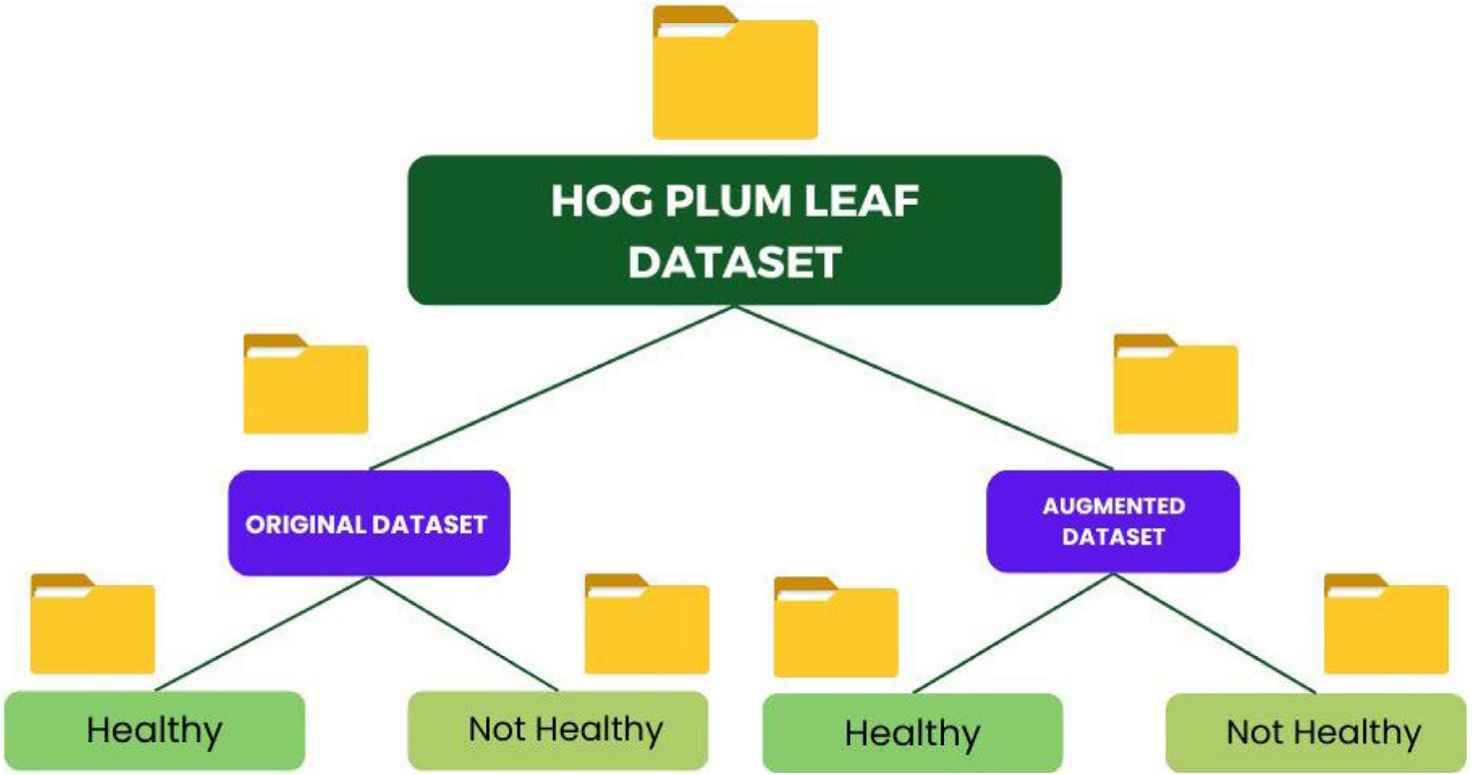
Dataset file organization of Hog Plum leaf disease.

In this respect, data augmentation became an important step toward the construction of robust and high-performance models for early and accurate detection of leaf diseases in support of precision agriculture that will help farmers to make timely and efficient disease management practices (Fig. 4).

### 5.3 Dataset structure

The dataset is organized into a clear folder hierarchy to help with easy navigation and usage. The folder structure is as follows:

The dataset is organized into two main directories**: Original_Dataset** and **Augmented_Dataset**, each containing subfolders for healthy and diseased leaves. The Original_Dataset folder includes the original images of Hog Plum leaves in their natural states. Within this folder, the Healthy subfolder contains images of healthy leaves, while the Not_Healthy subfolder holds images of diseased leaves.

The Augmented_Dataset folder contains images generated through various augmentation techniques, such as flipping, rotation, scaling, and other transformations, to increase the dataset's diversity for model training. Similar to the original dataset, the Augmented_Dataset is organized into two subfolders: Healthy (for augmented healthy leaf images) and Not_Healthy (for augmented diseased leaf images).

This structure ensures a clear distinction between the original and augmented datasets, making it easier to navigate and use the data for training and evaluation purposes.

## Limitations

The limitations of the research include the following: First, geographic representations are limited to Bangladesh, so generalizing the performance of the models could not be assured over different regions. Due to the quality of images varying from the environment and lighting, model consistency may get affected in real-world applications.

## Ethics Statement

We ensure that our study was conducted in full compliance with ethical guideline. No harm was caused to plants, animals, or humans. No data was sourced from social media platforms. All authors confirm adherence to ethical standards required for publication in Data in Brief.

## CRediT Author Statement

**Sabbir Rahman Durjoy:** conceptualization, methodology, writing original draft, data curation. **Md. Emon Shikder:** conceptualization, visualization, data curation, writing original draft, validation. **Mayen Uddin Mojumdar:** supervision, formal analysis, writing - review & editing.

## Data Availability

Mendeley DataHog Plum Leaf Disease Detection Dataset (Original data).
